# Natural Language Metaphors Covertly Influence Reasoning

**DOI:** 10.1371/journal.pone.0052961

**Published:** 2013-01-02

**Authors:** Paul H. Thibodeau, Lera Boroditsky

**Affiliations:** 1 Stanford University, Department of Psychology, Stanford, California, United States of America; 2 Trinity University, Department of Psychology, San Antonio, Texas, United States of America; Hungarian Academy of Sciences, Hungary

## Abstract

Metaphors pervade discussions of social issues like climate change, the economy, and crime. We ask how natural language metaphors shape the way people reason about such social issues. In previous work, we showed that describing crime metaphorically as a beast or a virus, led people to generate different solutions to a city’s crime problem. In the current series of studies, instead of asking people to generate a solution on their own, we provided them with a selection of possible solutions and asked them to choose the best ones. We found that metaphors influenced people’s reasoning even when they had a set of options available to compare and select among. These findings suggest that metaphors can influence not just what solution comes to mind first, but also which solution people think is best, even when given the opportunity to explicitly compare alternatives. Further, we tested whether participants were aware of the metaphor. We found that very few participants thought the metaphor played an important part in their decision. Further, participants who had no explicit memory of the metaphor were just as much affected by the metaphor as participants who were able to remember the metaphorical frame. These findings suggest that metaphors can act covertly in reasoning. Finally, we examined the role of political affiliation on reasoning about crime. The results confirm our previous findings that Republicans are more likely to generate enforcement and punishment solutions for dealing with crime, and are less swayed by metaphor than are Democrats or Independents.

## Introduction

Modern societies are faced with intractable social problems like crime, poverty, and climate change. How do we reason about such difficult multifaceted problems and create good social policies? The complexity and amount of information relevant for decision-making in many policy domains far exceeds what an average citizen (or even a seasoned policy wonk) can be expected to master and maintain in mind. To make matters worse, the voting public is, on average, strikingly uninformed about the workings of government and the details of social policy issues [1]–[2]. Nonetheless, citizens form policy preferences and express these preferences in their votes and campaign donations. How can average citizens get conceptual entree into social problems and make sense of complex policy issues?

One possibility presents itself in patterns in language. To discuss social and political issues, we rely heavily on metaphor [3]–[4]. Whether we’re discussing *hunting down* drug lords, *propping up* dictators, or trying to *jump-start* the economy, we are borrowing terms from everyday domains of knowledge (hunting, physical support, cars) to talk about complex social and political issues. Our propensity for metaphorical discussion of policy comes with both benefits and costs. Novel metaphors can lead us to think about old problems in new ways and to discover new solutions. Further, by simplifying the problem space, and allowing reuse of knowledge from everyday experience, metaphors can allow more people to participate meaningfully in policy discussion.

However, each metaphorical frame offers only a partial view of the problem space. Frames streamline information, necessarily selecting and organizing elements to simplify complex issues [5]. A given metaphor is able to accommodate only some aspects of the problem space and must exclude others. In some cases, such targeted framing may lead to an illusion of simplicity and bad policy decisions, even among experts. For example, writing about an economic stimulus bill proposed in the US Congress, economist Paul Krugman argued that the bill’s authors were misled by bad metaphors:

The deal, we’re told, will jump-start the economy; it will give a fragile recovery time to strengthen. I say, block those metaphors. America’s economy isn’t a stalled car, nor is it an invalid who will soon return to health if he gets a bit more rest. Our problems are longer-term than either metaphor implies. And bad metaphors make for bad policy. The idea that the economic engine is going to catch or the patient rise from his sickbed any day now encourages policy makers to settle for sloppy, short-term measures when the economy really needs well-designed, sustained support [6].

Given the ubiquitous nature of metaphor in social policy discussions, it is important to understand whether and how metaphors shape people’s reasoning about policy issues. In this paper we investigate the role of metaphor in reasoning about social policy in the domain of crime. Our studies are designed to further illuminate the mechanisms through which metaphors can shape understanding and reasoning. In particular we ask whether metaphorical framing can shape people’s decisions as they evaluate alternative solutions, and whether people need to be explicitly aware of metaphors to be influenced by them.

In previous work [7], we demonstrated that using different metaphors to talk about crime leads people to propose different solutions to addressing the crime problem. We focused on two contrasting metaphors for crime (crime as a virus and crime as a beast) and showed that these metaphors subtly encourage people to reason about crime in a way that is consistent with the entailments of the metaphors. In one study, we gave people a report about increasing crime rates in the city of Addison and asked them to propose a solution. For half of the participants, crime was metaphorically described as a beast preying on Addison, and for the other half as a virus infecting Addison. The rest of the report contained crime statistics that were identical for the two metaphor conditions. The results revealed that metaphors systematically influenced how people proposed solving Addison’s crime problem. When crime was framed metaphorically as a virus, participants proposed investigating the root causes of the problem and treating the community by enacting social reform by, for instance, eradicating poverty and improving education. When crime was framed metaphorically as a beast, participants took a much more direct approach in their proposals: catching and jailing criminals and enacting harsher enforcement laws.

In further studies, we modified the report to use only a single word to instantiate the metaphoric frame (“Crime is a virus/beast ravaging the city of Addison”). Even with this minimal one-word metaphorical intervention, we found that participants offered different problem solving suggestions, consistent with the metaphors. Further studies showed that these metaphorical framing effects result from people instantiating metaphor-consistent knowledge structures for representing the crime situation and cannot be explained by simple spreading activation among lexical associates.

However, this previous work leaves a number of key questions unanswered. First, in the previous studies, participants were asked to freely generate a solution to the crime problem. It is possible that metaphors make some solutions more available and so easier to bring to mind than others. This free-generation method may reveal what solution comes to mind first, but leaves open the question of whether metaphors influence what solution people think is best. When people make decisions about public policy in the real world, they don’t typically just generate one solution, but instead evaluate a number of competing proposals. It is possible that the effect observed in previous studies is an ephemeral effect at solution retrieval. Do metaphors affect people’s reasoning about social issues even if they are able to evaluate and compare a number of possible solutions?

Second, if metaphors affect reasoning, how explicit is this influence? Do people know they are being influenced by metaphors and do they need to be explicitly aware of the metaphor to be influenced by it? In previous work, we asked people to identify what part of the crime report was most influential in their reasoning. Very few people (around 5%) selected the metaphor. Instead, most people cited the crime statistics (which were the same in both conditions). However, this method allows for the possibility that people did think the metaphor was influential, but simply didn’t want to choose it – possibly because they wanted to appear rational in front of the experimenter. Citing statistics as influential allows one to appear objective. In new studies reported in this paper, instead of asking what part of the story was most influential, we tested participants’ memory for the metaphor. If participants can’t explicitly recall the metaphor, will they still show effects of the metaphorical frame in their reasoning about crime?

In Experiment 1, we first tested participants’ ability to explicitly extract the entailments of the virus and beast metaphors for crime. Participants were told that two politicians were advocating for different approaches to the crime problem, with one describing crime as a beast and one as a virus. We then asked participants to guess which crime-reducing solutions each politician might prefer from a set of possibilities. This study allows participants to explicitly compare the two metaphors. The results confirmed expectations from previous work. Participants associated the beast metaphor with solutions advocating enforcement and punishment, and associated the virus metaphor with solutions advocating social reform (improving the economy and educational system).

In Experiments 2–4, we tested whether participants would themselves be swayed by metaphors when evaluating proposed policies for reducing crime. Participants first read a report that framed crime as either a beast or a virus using a one-word metaphor (as in Experiment 2 of [7]). They then evaluated 4 or 5 policy proposals and indicated which they thought were best either by re-arranging them or dragging them into a response box in order of preference. Across these variations in method, the metaphorical frame mattered. Even when asked to select the best proposal from a set of alternatives, participants were influenced by the metaphorical frame. This suggests that metaphorical framing doesn’t only influence the ease with which people can retrieve a solution or which solution comes to mind first, but can also influence the evaluation stage - which solution people see as best.

In Experiments 2–4, we also investigated the extent to which people were using the metaphor explicitly to guide their reasoning about crime. In Experiment 2 we did this by asking people to indicate which part of the passage had been most influential in their decision. As before, we found that very few participants thought the metaphor played an important part in their decision. In Experiments 3 and 4 we asked people to recall the metaphor (given the surrounding context: “Crime is a _____ ravaging the city of Addison”). We found that participants who had no explicit memory of the metaphor were just as much affected by the metaphor as participants who were able to remember the metaphorical frame. These findings suggest that metaphors can act covertly in reasoning.

Finally, we examined the role of political affiliation on reasoning about crime. We confirmed previous findings that Republicans are more likely to endorse crime-reduction programs that emphasize enforcement and punishment, and are less swayed by metaphor than are Democrats or Independents.

## Methods

### Ethics Statement

The experiments reported here were done in accordance with the Declaration of Helsinki. Additionally, they followed the ethical requirements of the Stanford University institutional review board and complied with ethics guidelines set forth by the IRB recommendations; the Stanford University institutional review board reviewed and approved the protocol for studies presented here. Participants were informed that their data would be treated anonymously and that they could terminate the experiment at any time without providing any reason. We received written informed consent from all participants before they participated in an experiment.

### Participants

Participants in each of the four experiments were recruited and paid through Amazon’s Mechanical Turk (www.mturk.com). Turkers were paid $0.50 for their participation. Each experiment took about five minutes to complete. We used Mechanical Turk’s exclusion capabilities to ensure that participants lived in the United States and had an approval rating of 90% or better. This ensured that we sampled from a high quality pool of participants. We tracked participant identifiers (Mechanical Turk Worker IDs) to ensure that no one had participated in a previous version of a similar experiment or in more than one of the experiments reported here. Otherwise, we did not eliminate participants. We sought roughly 100 participants per condition in Experiments 1 and 3 and 200 participants per condition in Experiments 2 and 4.

#### Experiment 1

There were 226 people who participated in Experiment 1 in exchange for pay. Of these, 104 were female and 122 were male. Their ages ranged from 18 to 65 with a mean of 30.88 (median 25); 82 identified as Democrats, 104 as Independents, and 40 as Republicans.

#### Experiment 2

There were 415 people who participated in Experiment 2. Of these, 212 were female and 203 were male. Their ages ranged from 18 to 75 (mean = 31, median = 25); 155 participants identified as Democrats, 210 as Independents, and 50 as Republicans.

#### Experiment 3

Of the 171 Turkers in Experiment 3, there were 92 females and 79 males, whose ages ranged from 18 to 75 (mean = 31, median = 25). And of these, 77 identified as Democrats, 64 as Independents, and 30 as Republicans.

#### Experiment 4

Of the 353 Turkers in Experiment 4, there were 212 females and 141 males, whose ages ranged from 18 to 65 (mean = 34, median = 25). Of these, 134 identified as Democrats, 144 as Independents, and 75 as Republicans.

### Materials

In each of the four experiments, participants read a description of a crime problem in a fictional city, Addison. In Experiment 1, it read as follows:

Crime is ravaging the city of Addison. Five years ago Addison was in good shape, with no obvious vulnerabilities. Unfortunately, in the past five years the city’s defense systems have weakened, and the city has succumbed to crime. Today, there are more than 55,000 criminal incidents a year - up by more than 10,000 per year. There is a worry that if the city does not regain its strength soon, even more serious problems may start to develop.

The crime report used in Experiments 2–4 was nearly identical to that of Experiment 1. The only difference between them is that the report used in Experiments 2–4 included one of two metaphoric frames in the first sentence: “Crime is a {virus/beast} ravaging the city of Addison.”

In each of the four experiments, immediately after reading the crime report, participants were presented with four or five possible approaches to the crime problem. These approaches are listed below. The first four of the following five options were included in each of Experiments 1–4. Option 5, “neighborhood watches” was only included in Experiments 3 and 4. The options were always displayed in random order.

1. Increase street patrols that look for criminals.
2. Increase prison sentences for convicted offenders.
3. Reform education practices and create after school programs.
4. Expand economic welfare programs and create jobs.
5. Develop neighborhood watch programs and do more community outreach.

### Design

#### Experiment 1

In Experiment 1, participants first read the non-metaphorically framed crime report. After learning about the crime problem in Addison, they read that two city officials were engaged in a debate over how to reduce crime in the city, and that these two officials were using contrasting metaphors to support their message: one claimed that crime was a “virus” and the other that crime was a “beast.” They were told that there were four crime-reducing programs available to the city and their task was to guess which program each of the officials supported.

After submitting their responses, participants answered a series of background questions. These included questions about their age, gender, language history, educational background, geographic location, and political affiliation. Participants were asked to self-identify their political affiliation by answering the multiple choice question “What is your political affiliation?” Possible answers included options for “Republican,” “Democrat,” and “Independent.” Independents were asked a follow-up question about their ideology, “Would you describe yourself as relatively more conservative, liberal, or neither?” Possible answers for this question included options for “Conservative,” “Liberal,” or “Middle.”

#### Experiments 2–4

Experiments 2–4 were nearly identical versions of one another, using slightly different methods to elicit evaluations. Because the methods and results of the Experiments were so similar, we describe them together, pointing out differences as appropriate.

In all three experiments, participants first read the report about a worsening crime problem, with crime metaphorically framed as either a beast or a virus ravaging the city.

After reading the report, their task was to evaluate a set of programs designed to reduce crime in Addison and to select which would be most effective. In Experiments 2 and 3, participants indicated their preference by dragging and dropping the response options from a bank on the left side of the browser window to an empty text box on the right side of the browser window. In Experiment 4, participants indicated their preference by reordering the response options in place. In each Experiment, the crime report was not on the screen while participants were evaluating the response options; participants were not able to go back and re-read the report before making their selections.

On the subsequent screen, participants were asked either to identify the part of the report that was most influential in their suggestion or to try to remember the metaphor frame. In Experiment 2, participants identified the part of the report that was most influential to their suggestion by copying and pasting a section of the report into a text box. In Experiments 3 and 4, participants responded to a cued recall question: “The report you read started ‘Crime is a _____ ravaging the city of Addison.’ Please fill in the blank.”

After the experiment, participants answered a series of background questions. These included questions about their age, gender, language history, geographic location, and political affiliation as in Experiment 1. Participants also answered questions from three personality inventories: the BFI-10 [8]–[10], the Need for Cognition scale [11]–[12], and the Fascism Scale (f-scale) [13]. No other variables were measured.

#### Coding

The five crime-reducing suggestions were coded in two ways. First, crime-reducing suggestions were coded into two categories: “enforce” and “reform”. The degree to which each response emphasized enforcement (or reform) was established by asking a separate group of 35 people on Mechanical Turk to rate the five options on a single continuous dimension ranging from exclusively emphasizing social reform (0) to exclusively emphasizing enforcement (100).

The results of this norming study revealed a categorical distinction between the responses: people rated “street patrols” (*M* = 87.21, *sd* = 13.5), “prison sentences” (*M* = 85.11, *sd* = 22.37), and “neighborhood watches” (*M* = 58.69, *sd* = 25.77) as more enforcement- than reform-oriented; the reverse was true for “educational reform” (*M = *17.14, *sd* = 27.13), and “economic welfare” (*M* = 20.82, *sd* = 30.93). As a result, “street patrols,” “prison sentences,” and “neighborhood watches” were categorized as enforcement-oriented whereas “educational reform” and “economic welfare” were categorized as reform-oriented.

The “neighborhood watches” option was counted as enforcement-oriented because it was rated significantly above the midpoint of the scale, *t* [34] = 2.00, *p = *.05. However, the fact that this option was not rated as extreme as “street patrols” or “prison sentences” suggests that it may represent a more balanced approach. For this reason, we did not include this option in the response set in Experiments 1 or 2.

Second, responses were categorized as being “congruent” or “incongruent” with the metaphor. Consistent with previous work [7], “enforcement” responses were coded as congruent with the beast frame, and “reform” responses were coded as congruent with the virus frame.

## Results

### Experiment 1

In Experiment 1, we tested whether people would be able to explicitly extract the entailments of the virus and beast metaphors for crime. Participants in Experiment 1 where given both metaphor frames and were asked to associate one crime-reduction program with each. If the two metaphors lend themselves to different ways of conceptualizing a crime problem, and, if explicitly comparing the two metaphors helps bring these differences to mind, then we should expect people to associate enforcement-oriented programs with the beast metaphor and reform-oriented programs with the virus metaphor.

#### Enforcement versus Reform

Overall, suggestions that emphasized enforcement (63%) were numerically more popular than suggestions that emphasized reform. To test the significance of this trend, we numerically coded the degree to which participants selected enforcement- or reform-oriented programs to match with the metaphorical frames (as 2, −2, or 0, for selecting two enforcement-oriented programs, two reform-oriented programs, or one of each, respectively; we did not conduct a chi-square on these data because each participant contributed two non-independently sampled data points.). A t-test on this distribution (*M = *.53, *sd* = 1.02) confirmed that the enforcement-oriented suggestions were more popular overall, *t* [225] = 7.86, *p*<.001.

**Figure 1:**
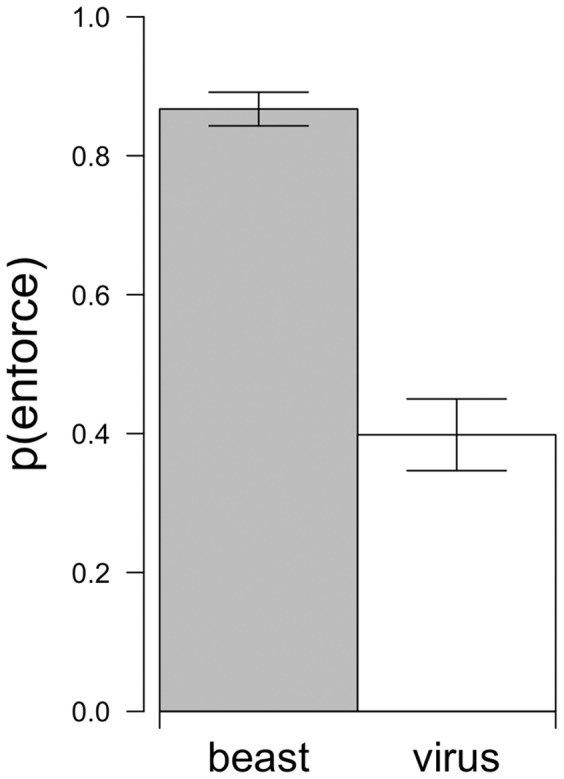
Explicit metaphor comparison. People in Experiment 1 were more likely to associate enforcement-oriented responses with the “beast” metaphor than the “virus” metaphor. Error bars represent the standard error of the proportions.

Responses that were matched to the beast frame were more likely to emphasize enforcement (87%); responses that were matched to the virus frame were not (40%) (see Figure 1). We test the statistical significance of this tendency to match crime-reduction responses congruently with metaphor frames in the section below.

#### Congruence

To test whether participants conceptualized crime differently on the two metaphors, we computed a single congruence value for each person. Participants could associate 0, 1, or 2 crime-reduction programs congruently with the two metaphor frames. By chance, one would expect an equal proportion of participants at each of these levels. Selecting a crime-reduction program to associate congruently with the first metaphor (*p* = 1/2) increases the likelihood of selecting a crime-reduction program to associate congruently with the second metaphor (*p* = 2/3). Therefore, the probability of submitting two congruent responses by chance is 1/3 (i.e., 1/2 * 2/3). Analogously, there is a 1/3 chance of submitting two incongruent responses by chance.

The results showed a tendency to congruently match responses with metaphors: 129 (57%) participants submitted two congruent responses, 74 (33%) submitted only one congruent response, and 23 (10%) submitted no congruent responses. This observed distribution was significantly different from what one would expect by chance, *χ* ^2^[2,N = 226] = 74.61, *p*<.001.

Of the 74 participants who gave only one congruent response, significantly more submitted two enforcement-oriented responses (91%) than two reform-oriented responses (9%), *χ* ^2^[1,N = 74] = 48.65, *p*<.001. This is evidence of a response bias towards enforcement.

These results suggest that people can extract the metaphorical entailments of the two metaphors when they have an opportunity to compare the two frames explicitly: participants associated the beast metaphor with solutions advocating enforcement and punishment, and associated the virus metaphor with solutions advocating social reform (improving the economy and educational system).

In Experiments 2–4, we extend this, and previous, findings in several ways. First, we test whether it is necessary to compare the two metaphors explicitly in order for them to lead people towards different opinions on solving crime (thereby replicating [7]). Second, we test whether the metaphors simply makes some response options more available in memory or whether they actually influence what crime-reduction programs people consider to be best. Finally, we test the degree to which the metaphor framing effect depends on deliberate consideration of the metaphor.

### Experiments 2–4

We found, first, that the influence of the metaphor did not differ across Experiments 2–4. A logistic regression model that included interaction terms between Experiment (‘2’, ‘3’, ‘4’) and frame (‘virus’ vs. ‘beast’) did not predict participants’ responses better than one that did not include interaction terms, *χ* ^2^ [2,933] = .54, *p = *.76. As a result, we have collapsed across the three experiments in presenting results.

#### Fighting Crime

The metaphor frame influenced what participants considered the best response to the crime problem in Addison. People who read that crime was a beast were more likely to rank one of the enforcement-oriented responses as the best (42%) than those who read that crime was a virus (31%). A logistic regression model that included a regressor for metaphor frame was a better fit to the data than one that did not, *χ* ^2^ [1,937] = 13.05, *p*<.001. A chi-square test of independence confirms that people who read the beast metaphor were more likely to suggest an enforcement-oriented solution, *χ* ^2^[N = 939] = 12.55, *p*<.001.

Of note, excluding data from participants who chose the ‘neighborhood watches’ option in Experiments 3 and 4 does not affect the results. A logistic regression reveals a significant effect of frame on response, *χ* ^2^ [1,854] = 13.94, *p*<.001; a chi-square test of independence confirms this effect, *χ* ^2^[N = 856] = 13.34, *p*<.001.

**Figure 2:**
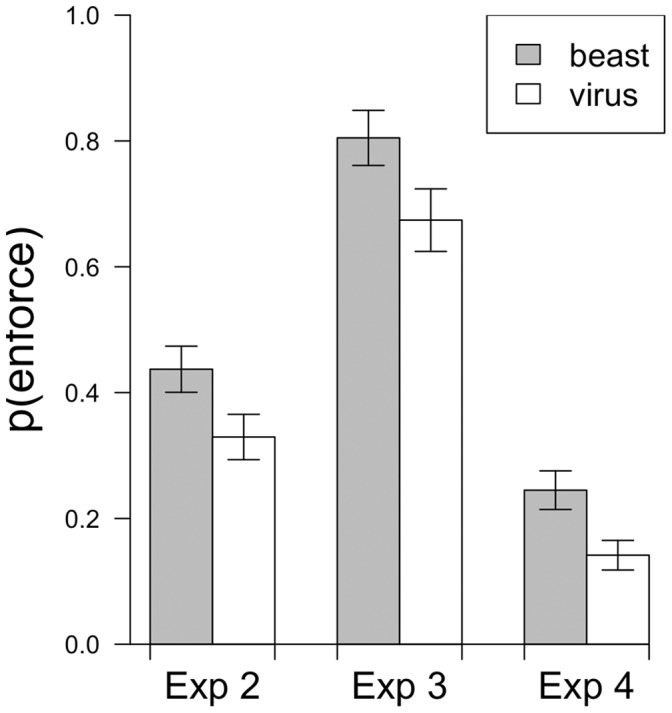
Natural language metaphors. In Experiments 2–4 people who read that crime was a beast were more likely to select an enforcement-oriented response to crime as their first choice than people who read that crime was a virus. Error bars represent the standard error of the proportions.

This pattern can be seen in each of the three experiments (see Figure 2). In Experiment 2, reading that crime was a beast made people 10% more likely to endorse an enforcement-oriented option (55% congruent), *χ* ^2^ [1,413] = 7.18, *p*<.01. In Experiment 3, reading that crime was a beast made people 13% more likely to endorse an enforcement-oriented option (57% congruent), *χ* ^2^ [1,169] = 3.90, *p*<.05. In Experiment 4, reading that crime was a beast made people 11% more likely to endorse an enforcement-oriented option (56% congruent), *χ* ^2^ [1,351] = 4.34, *p*<.05.

#### Metaphor covertness

We used two different methods to test whether people actively used the metaphor to reason about crime. In Experiment 2, participants were asked to identify the part of the report that influenced their suggestion by copying and pasting it into the response box. Eleven people (3%) included the metaphoric frame in their response. This small percentage of participants who identified the metaphor as influential is consistent with previous results [7].

In Experiments 3 and 4, participants were asked if they could remember the metaphoric frame in a cued recall task. Together, 246 participants (47% overall; 98 people, 57%, in Experiment 3 and 148 people, 42%, in Experiment 4) remembered the metaphor frame or a related synonym of the frame (e.g., we considered “disease” and “plague” as remembering the “virus” metaphor; similarly, we considered “mongrel,” “predator,” and “animal” as remembering the “beast” metaphor). People who could not remember the frame most often wrote “I don’t remember” or “problem” in the blank.

The substantial number of participants in both groups – “remember” and “forgot” – in Experiments 3 and 4 allowed us to test whether the metaphor frame only influenced people who remembered the metaphor. In fact, we found that people who remembered the metaphor (54% congruent) and people who forgot it (56% congruent) were both influenced by the frame, with no statistical difference between the two groups *χ* ^2^[1,N = 353] = .043, *p* = .84.

#### Variability in the main effect

Despite the consistency in the influence of the metaphor across Experiments 2–4, we found variability in the main effect – the proportion of responses that were coded as emphasizing enforcement – across the three experiments. In Experiment 2, 19% of responses were coded as emphasizing enforcement; in Experiment 3, 76% of responses were coded as emphasizing enforcement; and in Experiment 4, 39% of responses were coded as emphasizing enforcement. This difference across experiments was significant, *χ* ^2^[2,N = 939] = 157.57, *p*<.001.

There are two identifiable sources of variability in the main effect across the three experiments. The first relates to the specific set of response options that participants were asked to consider. In Experiments 3 and 4, participants considered five response options, three of which were coded as enforcement-oriented. In Experiment 2, participants considered only four response options, two of which were coded as enforcement-oriented. Adding the option that described a neighborhood watch program in Experiments 3 and 4 increased the likelihood that people chose an option that was coded as emphasizing enforcement (by 31%), *χ* ^2^ [1,938] = 100.07, *p*<.001. As described earlier, the “neighborhood watch” option was rated as considerably less enforcement oriented by an independent group of raters than the other two enforcement options (58.69% enforcement for “neighborhood watch” as compared to 87.21% and 85.11% enforcement for increasing street patrols and prison sentences respectively), so what appears as an increase in enforcement-oriented solutions in Experiments 3 and 4 is at least in part an artifact of including this extra, and more intermediate response option.

The second factor contributing to this variability relates to the individual characteristics of the participants in the three experiments. Participants in Experiments 3 and 4 were, on average, more conservative than participants in Experiment 2, *t* [937] = 1.96, *p*<.05, which may help to explain why there is an increase in the tendency to emphasize enforcement in Experiments 3 and 4.

#### Background measures

We found no effect of age, gender, language history, educational background, geographic location, or personality.

### Political Affiliation

Consistent with previous work [7], we found systematic differences in the response patterns of Democrats, Independents, and Republicans. In Experiments 2–4, Republicans were more likely to choose an enforcement-oriented response (55%) than Independents (33%) or Democrats (32%), *χ* ^2^ [1,934]* = *24.90, *p*<.001.

Further, Democrats and Independents were numerically more likely to be influenced by the frame than Republicans. In Experiments 2–4, after reading the beast metaphor, Democrats and Independents were 12% and 13% more likely to suggest an enforcement-oriented response, respectively, whereas Republicans were 3% less likely to suggest an enforcement-oriented response.

To investigate whether Republicans truly were less influenced by the metaphor frame, we pooled data from Experiments 2–4 reported here with Experiments 2 and 4 from [7]. Pooling data across the experiments (each of which uses the same crime report and similar dependent measures; and each of which yielded similar results: with 55–60% of responses congruent with the frame) affords the most stable and representative data for the purpose of this analysis.

In conducing this analysis, we fit a series of logistic regression models using congruence as the dependent measure. We found, first, that there was no difference in the effect across the five experiments: including separate regressors for each experiment did not improve the fit of the model, *χ* ^2^ [4,1314] = .91, *p = *.92.

**Figure 3:**
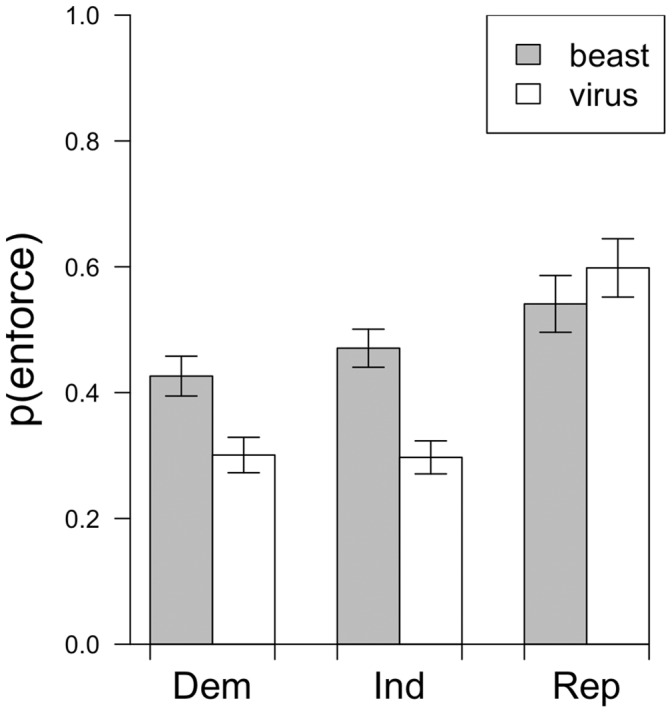
Political affiliation and metaphor persuasion. Democrats and Independents but not Republicans show a consistent influence of the metaphor frame across Experiments 2–4 and Experiments 2 and 4 from [7]. Error bars represent the standard error of the proportions.

In the second model, we added two additional regressors: one contrasted Republicans from non-Republicans (i.e., Democrats and Independents) and the other contrasted Democrats from Independents. We designed these contrasts to test whether the metaphor selectively influenced Democrats and Independents (and not Republicans), and, further, whether there was a difference in the influence of the metaphor between Democrats and Independents. Including these regressors did significantly improve the model, *χ* ^2^ [2,1316] = 9.59, *p*<.01. The results of the model revealed that the metaphor significantly predicted the responses of non-Republicans (relative to Republicans), *z = *2.97, *p*<.01. The non-Republicans (i.e., the Democrats and Independents) were similarly influenced by the metaphor, *z = *.81, *p = *.42 (see Figure 3).

Of note, the relatively small number of Republicans contributing data to Experiments 2–4 of this paper (*n* = 155, which represents 17% of participants) prevents us from having the power to detect this difference in these experiments alone. On such an analysis, including regressors to compare Republicans to non-Republicans and Democrats to Independents does not significantly improve the fit of a model applied to these data, *χ* ^2^ [2,936] = 2.91, *p* = *ns.* However, the regressor differentiating non-Republicans from Republicans approaches significance, *z = *1.71, *p = *.09, and the regressor differentiating Democrats from Independents did not approach significance, *z = *.02, *p = *.98, as in the combined analysis. In addition, there is no interaction between data set (current vs. previous) and either regressor (Rep vs. non or Dem vs. Ind), *χ* ^2^ [3,1316] = 3.86, *p* = *ns*. This suggests that a similar pattern exists in the previous and current data sets.

There are several reasons why Republicans may be less influenced by the metaphor frame than Democrats and Independents. Republicans may simply be resistant to framing in general or metaphor framing specifically. This is unlikely as others have found an effect of a framing manipulation on Republicans. Hardisty, Johnson, and Weber [14], for instance, found that when a carbon-reduction program was labeled as an offset rather than a tax, Republicans were much more likely to support it.

More likely, Republicans may be resistant to persuasion on the issue of crime, just as Democrats may be resistant to framing on an issue to which they are ideologically committed. Indeed, Hardisty, Johnson, and Weber [14] found that while Republicans’ opinions on an environmental cost were subject to change on a framing manipulation, Democrats’ were committed to carbon-reduction regardless of whether it was labeled as a tax or offset.

## Discussion

In four experiments we investigated the role of metaphor in how we conceptualize and reason about complex policy issues like crime. In Experiment 1, we asked people to explicitly compare two metaphors for crime and to evaluate whether they seemed to support different crime-reduction programs. We found that deliberately comparing the metaphors allowed people to infer metaphor-consistent approaches to the crime problem: a virus metaphor for crime was associated with more systemic, reform-oriented approaches to crime reduction whereas a beast metaphor for crime was associated with more direct, enforcement-oriented approaches.

In Experiments 2–4 participants read one of these two metaphors embedded in a larger description of a crime problem. In these experiments participants were not instructed to use the metaphors deliberately, nor were they presented with multiple, contrasting frames. Nevertheless, we found that the metaphors influenced how people conceptualized and reasoned about the problem in a way that was consistent with the results of Experiment 1. Further, we found that people rarely identified the metaphor as influential in their thinking despite its influence. Indeed, in Experiments 3 and 4, we found that the metaphors influenced even those people who could not remember the metaphorical frame. Metaphors can instantiate coherent knowledge-structures that influence how we build a representation of the problem and evaluate potential solutions, and they can do so even when they slip by unnoticed.

Metaphorical frames can play a powerful role in reasoning because they implicitly instantiate a representation of the problem in a way that steers us to a particular solution. Because social policy issues are multi-faceted and complex, metaphorical frames may be particularly seductive in policy reasoning. In a large and underdetermined problem space, it may be difficult to evaluate or falsify a metaphorical frame or to notice what aspects of the problem the frame might exclude. And since metaphorical frames necessarily select and streamline information into a conceptual structure, they may offer welcome relief from the cognitive complexity inherent to social policy issues. This conceptual structuring may lead to new solutions, but it also means that reasoners may be less likely to be on guard for or resistant to the conceptual structures suggested in metaphors.

Our work shows that people can be unwittingly swayed by metaphors when reasoning about social policy. Metaphors encourage particular conceptualizations of problems and, depending on the situation, can be helpful or misleading. We hope that coming to appreciate the role that metaphors play in reasoning can help decision-makers be mindful of the limitations and the virtues of the metaphors they chose to frame issues.
